# Mice Deficient in Cryptochrome 1 (*Cry1*^−/−^) Exhibit Resistance to Obesity Induced by a High-Fat Diet

**DOI:** 10.3389/fendo.2014.00049

**Published:** 2014-04-09

**Authors:** Guy Griebel, Christine Ravinet-Trillou, Sandra Beeské, Patrick Avenet, Philippe Pichat

**Affiliations:** ^1^Exploratory Unit, Sanofi R&D, Chilly-Mazarin, France; ^2^Infectious Diseases Therapeutic Strategic Unit, Sanofi R&D, Toulouse, France

**Keywords:** clock genes, cryptochromes, obesity, diet-induced obesity, knockout mice, mice, triglycerides

## Abstract

Disruption of circadian clock enhances the risk of metabolic syndrome, obesity, and type 2 diabetes. Circadian clocks rely on a highly regulated network of transcriptional and translational loops that drive clock-controlled gene expression. Among these transcribed clock genes are cryptochrome (CRY) family members, which comprise *Cry1* and *Cry2*. While the metabolic effects of deletion of several core components of the clock gene machinery have been well characterized, those of selective inactivation of *Cry1* or *Cry2* genes have not been described. In this study, we demonstrate that ablation of *Cry1*, but not *Cry2*, prevents high-fat diet (HFD)-induced obesity in mice. Despite similar caloric intake, *Cry1*^−/−^ mice on HFD gained markedly less weight (−18%) at the end of the 16-week experiment and displayed reduced fat accumulation compared to wild-type (WT) littermates (−61%), suggesting increased energy expenditure. Analysis of serum lipid and glucose profiles showed no difference between *Cry1*^−/−^ and WT mice. Both *Cry1*^−/−^ and *Cry2*^−/−^ mice are indistinguishable from WT controls in body weight, fat and protein contents, and food consumption when they are allowed unlimited access to a standard rodent diet. We conclude that although CRY signaling may not be essential for the maintenance of energy homeostasis under steady-state nutritional conditions, *Cry1* may play a role in readjusting energy balance under changing nutritional circumstances. These studies reinforce the important role of circadian clock genes in energy homeostasis and suggest that *Cry1* is a plausible target for anti-obesity therapy.

## Introduction

The biological clock is an extensive molecular network that provides circadian time-keeping, controlling and maintaining daily rhythms of many behavioral and physiological processes. In mammals, it comprises a complex circuitry of transcriptional and translational regulatory feedback loops, including the core transcriptional activators CLOCK and BMAL1, which activate expression of three *Period* (*Per1–3*) and two *cryptochrome* (*Cry1* and *Cry2*) genes (1). These latter products are part of the negative regulatory arm of the circadian clock system as their rhythmical accumulation leads to the formation of a repressor complex that interacts with CLOCK and BMAL1 to inhibit their own transcription. Mammalian clocks not only reside in the suprachiasmatic nucleus (SCN), in which the clock is determined by light signals through the retinohypothalamic tract, but they have been identified in a wide array of peripheral organs including heart, lung, kidney, liver, and pancreas, where their timing is set by metabolic cues (2–4).

Perturbation of endogenous circadian rhythms driven by clock gene mutations or disruptive lifestyles has been shown to give rise to various pathophysiological manifestations (5–7). This association is particularly strong with respect to cancer and metabolic disease. For example, sleep disturbances or long-term shift work has been shown to increase the risk of developing type 2 diabetes (8–11). Moreover, polymorphisms of the core clock genes *Clock* and *Bmal1* are associated with obesity and type 2 diabetes (12, 13). Genetic mouse models also show that mutation in the genes encoding core clock transcription factors resulted in metabolic disturbances [for a review, see Ref. (6)]. This is best illustrated by mice deficient in *Clock* or *Bmal1*, which display impaired glucose tolerance and reduced insulin secretion (14). Moreover, disruption of *Per2* and *Per1–3* in mice results in increased vulnerability to high-fat-induced obesity and/or food intake (15). However, the situation is far from being clear with respect to these repressor clock genes as other studies demonstrated that mutation in *Per 1, Per2*, and *Per1–3* resulted in reduced food intake and/or body weight in mice (16, 17). It was argued that the PER proteins may not be the best representatives of the negative regulatory arm of the molecular clock because they do not directly inhibit the core clock proteins, unlike cryptochromes (CRY) (18). Although there are several studies on the consequences of *Cry1, Cry2*, and *Cry1/2* deletion, mainly on behavioral and molecular rhythmicity, only one has investigated the effects of *Cry1*^−/−^*Cry2*^−/−^ double mutation on metabolism (18). These authors showed that deficiency in CRY resulted in increased susceptibility to diet-induced obesity as a consequence of increased insulin secretion and lipid storage.

To explore further the role of CRY on metabolism, the current study was undertaken to assess the effects of single deletion of either *Cry1* or *Cry2* on high-fat-induced obesity.

## Materials and Methods

### Animals

In all experiments mice were housed individually at a constant temperature of 21 ± 1°C and humidity (50 ± 10%) on a reverse light–dark cycle under a 12:12 light/dark cycle (light on at 7:00 a.m.). Six- to 10-week-old male *Cry1*^−/−^, *Cry2*^−/−^ mice, and their respective wild-type (WT) counterparts were obtained from The Jackson Laboratories. They were fed *ad libitum* either under standard-mouse diet (STD) containing 3.7 kcal/g (A04, UAR; 15% fat, 12% protein, 73% carbohydrate) or under a high-fat diet (HFD) of 4.7 kcal/g energy density (D12451, Research Diet; 45% fat, 22% protein, 33% carbohydrate). Genotypes were determined by PCR of tail DNA. Animals were age-matched WT controls (*Cry1*^+/+^ or *Cry2*^+/+^) and homozygous (*Cry1*^−/−^ or *Cry2*^−/−^) offsprings produced by backcrossing onto C57BL/6J background. All experimental procedures described herein were approved by the Animal Care and Use Committee of Sanofi or the Institutional Animal Care. Our animal facilities and animal care and use programs are in accordance with French legislation which implemented the European directive 86/609/EEC.

### Experimental procedure

In the first experiment, 8- to 10-week-old *Cry1*^−/−^ mice (*n* = 9–10) were compared to WT animals (*Cry1*^+/+^) in two feeding paradigms for 16 weeks: (a) on a STD laboratory regimen, and (b) on a HFD regimen. In the second experiment, 6- to 9-week-old *Cry2*^−/−^ mice (*n* = 12) were compared to their WT counterparts (*Cry2*^+/+^) (*n* = 15) in the two same feeding paradigms for 10 weeks. We have used a shorter time period in the *Cry2* study because body weight evolution was not different between groups after 2 months of experiment, regardless the genotype and the regimen.

### Body weight, body composition, and energy intake

Body weight changes were recorded once a week. Body composition (fat and protein contents) was assessed in *Cry1*^+/+^ and *Cry1*^−/−^ mice fed a HFD at the end of the experiment using the total body electrical conductivity technique (TOBEC system, EMSCAN 3000). This instrument measures total body electrical conductivity of small animals in a non-invasive manner (19). Body composition expressed as percentage protein mass or fat mass was obtained mathematically: protein (a): [0.816662 + 0.002615 × TOBEC + 0.046624 × body weight (BW) − 0.000066 × BW × TOBEC]^2^; fat (b): (−0.732127 − 0.008899 × TOBEC + 0.198538 × BW)^2^. Because we observed a different evolution in weight gain in *Cry1*^−/−^ mice compared to their WT littermates under a HFD, energy intake was assessed weekly between weeks 12 and 16 in these groups.

#### Serum analysis

Plasma levels of glucose, triglycerides (TGs), free fatty acids (FFAs), and free cholesterol were determined in *Cry1*^+/+^ and *Cry1*^−/−^ mice at the end of the 16-week HFD regimen in fed animals. Animals were sacrificed, blood was collected into EDTA-rinsed capillaries, and plasma was immediately prepared. Free cholesterol, TGs, and glucose were determined using enzymatic kits from Sigma. Plasma FFA levels were measured using the Wako kit (Wako Chemicals, Richmond, VA, USA).

### Statistical analyses

All data are shown as mean ± SEM. ANOVA and *post hoc* analyses were performed using SAS v.12 software. For body weight changes, statistical analysis was performed using a two-way ANOVA (factor strain × diet, with repeated measures on factor week). For food intake, a two-way ANOVA (factor strain or diet, with repeated measures on factor week) was performed. Finally, for body composition and serum analyses, a one-way ANOVA was used followed by *post hoc* comparisons with the Dunnett’s test. *P*-values <0.05 were considered statistically significant.

## Results

### Susceptibility of *Cry1*^−/−^ mice to high-fat-induced obesity

#### Growth and body temperature

*Cry1*-deficient mice displayed the same fur quality than WT animals and they grew normally as assessed by naso-anal length, similar in both groups at 26-week of age (*Cry1*^+/+^ = 9.7 ± 0.1 cm; *Cry1*^−/−^ = 9.3 ± 0.2 cm). In addition, there were no significant differences detectable in the body temperature between the two genotypes (*Cry1*^+/+^-HFD: 38.3 ± 0.2°C; *Cry1*^−/−^-HFD: 38.0 ± 0.2°C).

#### Body weight

**Figure 1:**
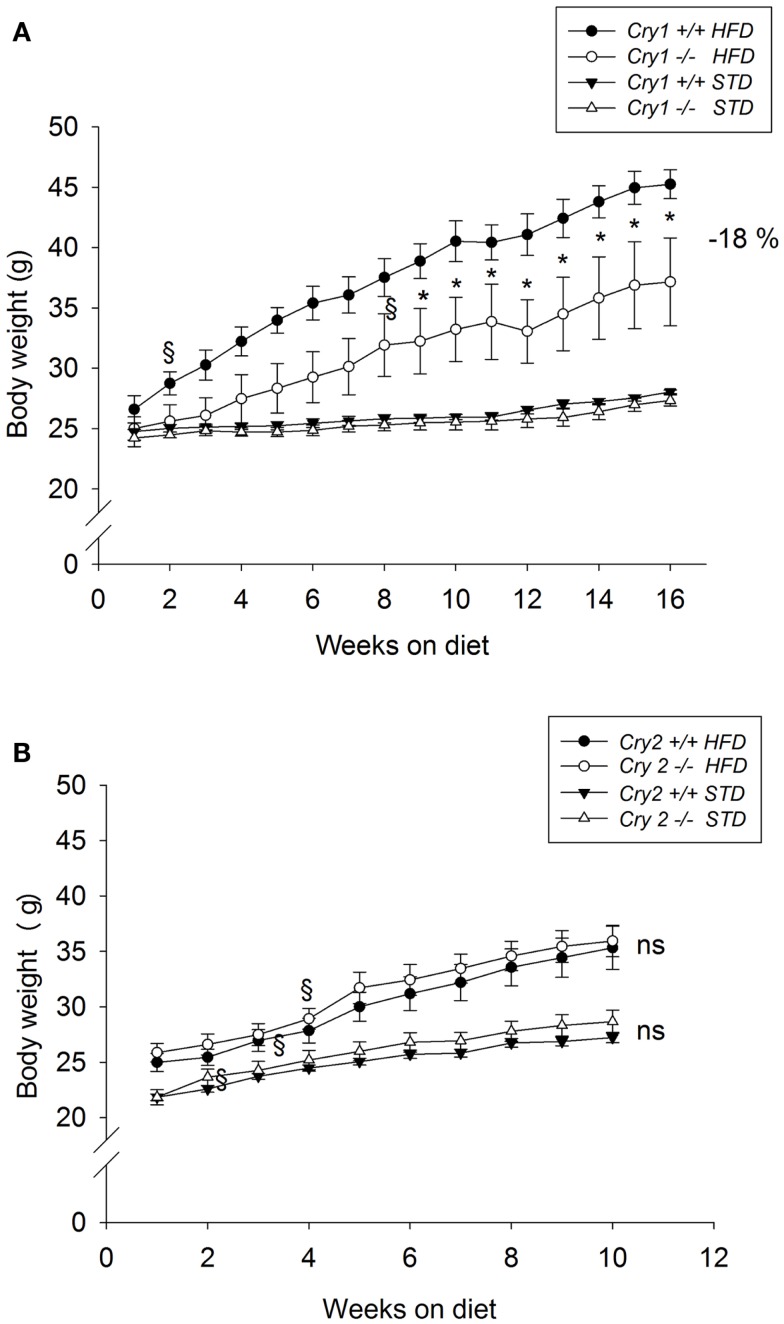
**Growth curves of WT control, Cry1-, and Cry2-deficient mice under STD and HFD**. Body weight for age-matched controls **(A)**: *Cry1*^+/+^ (filled symbols) and *Cry1*^−/−^ (open symbols) mice, **(B)**: *Cry2*^+/+^ (filled symbols) and *Cry2*^−/−^ mice (open symbols), determined once a week, on STD (triangles) and HFD (circles) regimens. Cry1 group: *n* = 5 (*Cry1*^+/+^-STD) and 5 (*Cry1*^−/−^-STD) and *n* = 4 (*Cry1*^+/+^-HFD) and 5 (*Cry1*^−/−^-HFD); Cry2 group: *n* = 6 (*Cry2*^+/+^-STD) and *n* = 7 (*Cry2*^−/−^-STD), and *n* = 8 (*Cry2*^+/+^-HFD) and *n* = 6 (*Cry2*^−/−^-STD). *P < 0.05, ns *P* > 0.05 indicated for genotype comparison, two-way ANOVA for body weight with repeated measures on factor week. ^§^*P* < 0.05, one-way ANOVA followed by Dunnett’s test for comparison with week 1 with repeated measures for parameter weight. Values shown are mean ± SEM.

**Figure 2:**
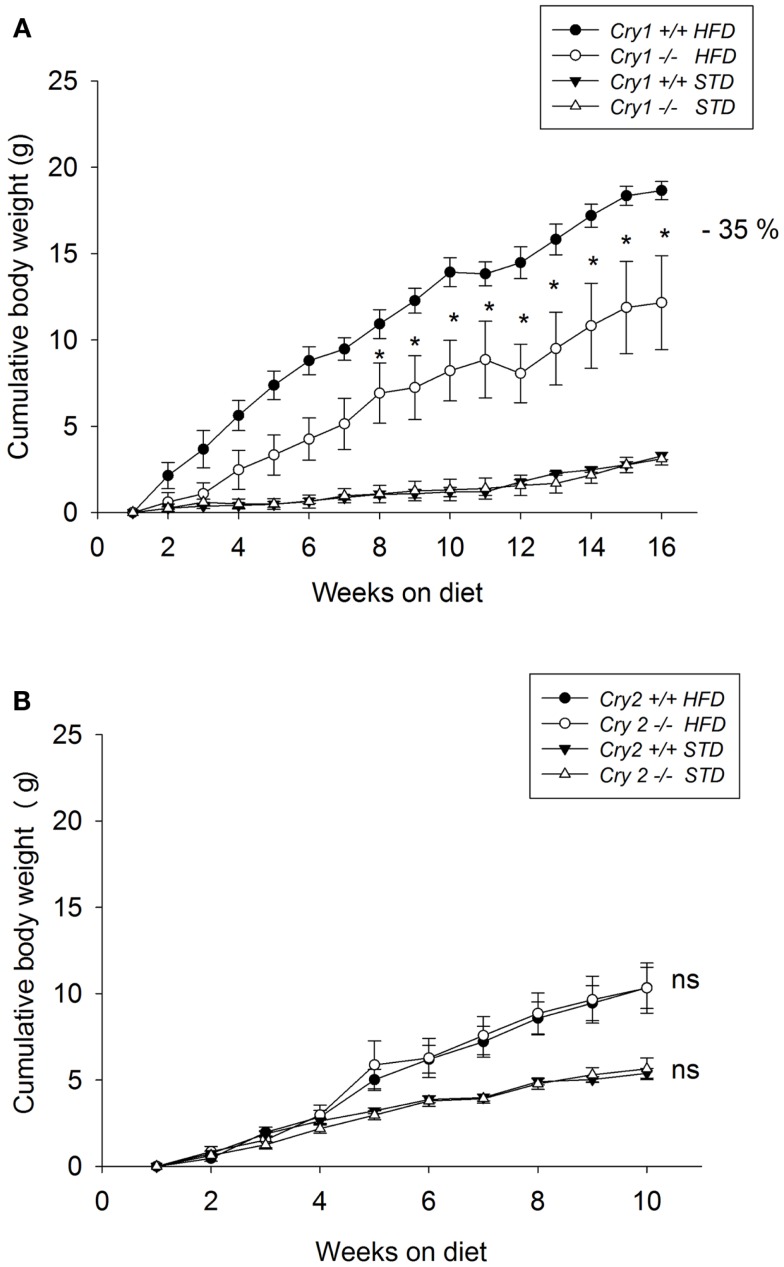
**Cumulative weight gain as calculated from mean weights of WT control, *Cry1-*, and *Cry2*-deficient mice under STD and HFD**. **(A)** *Cry1*^+/+^ (filled symbols) and *Cry1*^−/−^ (open symbols) mice, **(B)**: *Cry2*^+/+^ (filled symbols) and *Cry2*^−/−^ mice (open symbols), determined once a week, on STD (triangles) and HFD (circles) regimens. Cry1 group: *n* = 5 (*Cry1*^+/+^-STD) and 5 (*Cry1*^−/−^-STD) and *n* = 4 (*Cry1*^+/+^-HFD) and 5 (*Cry1*^−/−^-HFD); Cry2 group: *n* = 6 (*Cry2*^+/+^-STD) and *n* = 7 (*Cry2*^−/−^-STD), and *n* = 8 (*Cry2*^+/+^-HFD) and *n* = 6 (*Cry2*^−/−^-STD). *P < 0.05, ns *P* > 0.05 indicated for genotype comparison, two-way ANOVA for body weight with repeated measures on factor week. Values shown are mean ± SEM.

Body weight was not significantly different between mutant mice and WT animals when the feeding trials started (*Cry1*^+/+^ = 25.6 ± 0.6 g; *Cry1*^−/−^ = 24.6 ± 0.4 g). When maintained on a STD for 16 weeks, body weight changes did not differ significantly between *Cry1*^+/+^ and *Cry1*^−/−^ mice (Figure 1A). After 16 weeks on the HFD, *Cry1*^−/−^ mice showed a significantly lower body weight (37.2 ± 1.2 g, *P* < 0.001) compared with WT mice (45.3 ± 3.6 g). This effect reached statistical significance from week 8 [three-way ANOVA: *F*(15, 225) = 3.09, *P* < 0.001]. The total weight gain during the feeding period (Figure 2A) for *Cry1*^−/−^ mice on HFD was 12.2 g (+52%), whereas the WT mice on HFD gained 18.7 g (+70%) [three-way ANOVA: *F*(14, 210) = 2.43, *P* < 0.01].

#### Food intake

Analysis of food intake of animals on HFD shows that both WT and *Cry1*^−/−^ mice had similar mean energy intake ranging from 13.0 ± 0.4–16.7 ± 0.5 to 11.7 ± 0.7–16.1 ± 0.6 kcal/day (data not shown).

#### Body composition

**Figure 3:**
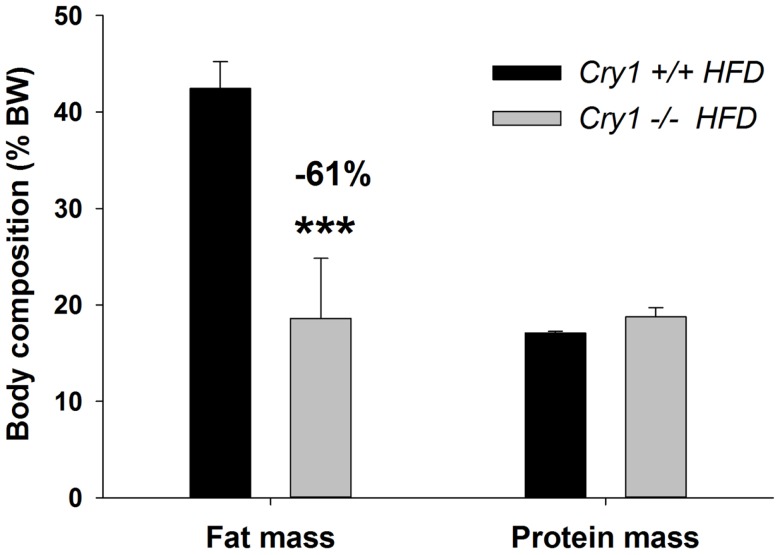
**Body composition (% BW) of WT control and *Cry1*-deficient mice**. Fat and protein masses (% body weight) for WT control *Cry1*^+/+^ (*filled bars*) and *Cry1*^−/−^ (*open bars*)-deficient mice, determined on a HFD regimen. Cry1 group: *n* = 4 (*Cry1*^+/+^-STD) and 5 (*Cry1*^−/−^-STD) and *n* = 8 (*Cry1*^+/+^-HFD) and 6 (*Cry1*^−/−^-HFD). One-way ANOVA for body composition. ***P < 0.001 significant difference for body composition of each strain. Values shown are mean ± SEM.

Analysis of body content showed that *Cry1*^−/−^ had reduced fat mass compared to WT animals (−61%) at the end of the 16-week HFD regimen [*F*(1, 7) = 12.34, *P* < 0.001], while they had similar lean mass accounting to 17.1 ± 0.2% (WT) and 18.8 ± 1.0% (*Cry1*^−/−^) of total body mass (Figure 3).

#### Lipid and glucose profiles

Because changes in weight are often associated with shifts in glucose and lipid metabolism, we surveyed serum levels of glucose, TGs, and FFAs. Tests were performed on mice that were maintained on HFD diet. Results revealed no difference in any of these parameters between WT and *Cry1*^−/−^ mice (Table 1).

**Table 1:** **Plasma lipids and glycemia in fed WT control and *Cry1*^−/^^−^ mice under HFD**.

Serum parameters (concentration)	WT	*Cry1*^−/−^
Triglycerides (mg/dl)	80.7 ± 13.2	97.4 ± 16.1
Free cholesterol (g/l)	2.05 ± 0.06	2.10 ± 0.1
Free fatty acid (mmol/ml)	0.95 ± 0.10	0.83 ± 0.15
Glucose (g/l)	2.00 ± 0.09	1.90 ± 0.10

*Values shown are mean ± SEM. WT, wild-type; HFD, high-fat diet. Shown are basal plasma lipids concentration, triglycerides (mg/dl), free cholesterol (g/l), free fatty acid (mmol/ml), and basal glycemia (g/l) in WT control *Cry1*^+/+^ and *Cry1*^−/−^ mice, determined at week 16 (*n* = 4)*.

### Susceptibility of *Cry2*^−/−^ mice to high-fat-induced obesity

#### Growth and body temperature

*Cry2*-deficient mice displayed the same fur quality than WT animals and they grew normally. Naso-anal length was similar in both groups at 15-week of age (*Cry2*^+/+^ = 9.1 ± 0.2 cm; *Cry2*^−/−^ = 9.1 ± 0.1 cm). Neither were significant differences in the body temperature between the two genotypes observed (*Cry2*^+/+^-STD: 37.2 ± 0.2°C; *Cry2*^−/−^-STD: 37.4 ± 0.2°C).

#### Body weight

Body weight was not significantly different between mutant mice and WT animals when the feeding trials started (*Cry2*^+/+^ = 23.5 ± 0.6 g; *Cry2*^−/−^ = 23.9 ± 0.8 g). When maintained on a STD for 10 weeks, body weight changes did not differ significantly between *Cry2*^+/+^ and *Cry2*^−/−^ mice (Figure 1B). After 10 weeks on the HFD, *Cry2*^−/−^ mice showed similar body weight changes (34.9 ± 1.1 g) than WT mice (34.4 ± 1.8 g). The total weight gain during the feeding period for *Cry2*^−/−^ mice on HFD was 10 g (+39%), whereas the WT mice on HFD gained 10.3 g (+41%) (Figure 2B).

Inasmuch as there were no weight change differences between *Cry2*^+/+^ and *Cry2*^−/−^ under both STD and HFD regimens at week 10, the experiment was discontinued and no additional metabolism parameters were assessed in these mice.

## Discussion

Our data provide several new insights into the physiological functions of CRY in mice. Utilizing *Cry1*^−/−^ and *Cry2*^−/−^ mice, we demonstrate for the first time that *Cry1*^−/−^ loss interferes with HFD-induced obesity, and that the physiological basis of this effect is not related to reduced energy intake. Both *Cry1*- and *Cry2*-deficient mice show no obvious phenotypical changes when fed a standard high-carbohydrate diet. The animals grow to WT-like size with normal body weights.

Male WT mice that consume a HFD achieve a body weight between 41 (Cry2 group) and 70% (Cry1 group) greater than that of littermates-fed STD and increased adipose tissue mass. HFD are frequently used in rodent models, including C57BL/6 mice (20, 21), to cause obesity associated with metabolic disturbances such as hyperglycemia, hyperinsulinemia, and non-alcoholic fatty liver disease. These phenotypic impairments grossly mimic abdominal obesity phenotypes in humans and are linked to the development of type 2 diabetes (22). In the current study, mice lacking *Cry2* did not display any overt phenotypical changes on a HFD. This was in contrast to *Cry1*^−/−^ mice fed under the same regimen, which showed a markedly reduced weight gain and reduced body fat stores compared with WT counterparts. Significant differences in body weight development generally arise from differences in energy intake and/or energy excretion. However, in the current study, we observed that food intake was not significantly decreased in *Cry1*^−/−^ mice under HFD conditions, suggesting increased energy expenditure. Body composition analysis revealed that *Cry1* deficit resulted in reduced fat content, while that of protein was not affected. Moreover, analysis of serum lipid and glucose profiles showed no difference between *Cry1*^−/−^ and WT mice at the end of the 16-week HFD regimen.

Deficiency of *Cry1* and/or *Cry2* has been shown to impair autonomic nervous system activity by increasing sympathetic neural outflow during the light phase, thus activating brown adipose tissue and hypermetabolism (23). It can be hypothesized that the increase in energy expenditure and resistance to HFD observed in the current study in *Cry1*^−/−^ would be consistent with increased sympathetic activity. Alternatively, it can be argued that the metabolism profile of *Cry1*^−/−^ mice may be the result of increased locomotor activity in these mice. While this parameter was not assessed here, it is worth mentioning that several previous studies on *Cry1*^−/−^ mice reported no difference in motor behavior between these mutant animals and their WT counterparts when tested under LD12:12 as was the case in our study (23, 24).

The metabolism phenotype of *Cry1*-deficient mice in the current study contrasts with that recently observed with *Cry1*^−/−^*Cry2*^−/−^ double mutant mice (18). In this study, mutant mice were reported to be lean under standard diet and when challenged with HFD, showed reduced food intake. Despite this hypophagia, they rapidly gained weight. The authors of this study suggested that hyperinsulinemia and increased lipid storage might account for the increased vulnerability of this mice to HFD-induced obesity. The discrepancy in metabolism phenotype between single and double mutant *Cry* mice cannot readily be attributed to methodological differences since the high-fat regimen used in both studies was the same (45% KJ fat). Moreover, background (C57BL/6J) and age range (8- to 10-week-old) of the mice also did not differ between these studies. It cannot completely be excluded that this difference is produced by a multitude of, perhaps small, methodological differences that do not necessarily become clear, even with close scrutiny of published reports. It is striking that in the Barclay et al.’s (18) study, HFD did not induce obesity in WT control mice, which is rather surprising since HFD is frequently used in rodent models, including C57BL/6 mice, to cause obesity associated with metabolic disturbances such as hyperglycemia, hyperinsulinemia, and non-alcoholic fatty liver disease (20, 21).

The apparent resistance of *Cry1*^−/−^ mice to weight gain with a HFD may seem counterintuitive, since the metabolic consequences of circadian disruption such as those observed in human clinical studies in shift workers or in genetic models involving clock gene ablation are generally associated with increased body weight and onset of metabolic syndrome (25–30). For example, mutant mice of the circadian gene Clock overeat become obese and develop hyperglycemia and dyslipidemia (31). These animals develop the adipocyte hypertrophy and excessive accumulation of fat in the liver that are hallmarks of the metabolic syndrome. Deletion of the three circadian period genes in mice cause increased weight gain on HFD (32). Similarly, *Bmal1*-deficient mice display increased fat deposition, elevated TGs/FFA levels, and disrupted insulin responsiveness (33–37). However, other studies targeting more selectively the negative arm of the clock have shown that *Per1* or *Per2* deficiency leads to reduced body weight and fat composition associated with a decrease in plasma levels of TGs and FFAs. These changes could not be attributed to differences in food intake or aberrant circadian regulation, but were suggested to be the result of increased energy expenditure (16, 17). To explain the metabolic effects of *Per2* ablation in this study, it was demonstrated that this clock gene modulates peroxisome proliferator-activated receptor γ2 (PPARγ2), a master regulator of adipogenic differentiation and lipid metabolism.

It is unclear whether a comparable scenario (i.e., *Cry1* controlling the activity of proteins critical for adipogenesis by operating as their natural modulators) can explain the current observed metabolism profile in *Cry1*^−/−^ mice. To the best of our knowledge, CRYs have not been described to control lipid metabolism by regulating transcriptional activity of relevant proteins. However, they were shown to participate in the regulation of hepatic gluconeogenesis notably through CRY-mediated inhibition of cAMP-mediated phosphorylation of cAMP response element-binding protein (CREB) (38–40). In particular, it was shown that fasted *Cry1* and *Cry2* double KO mice displayed increased expression of gluconeogenic genes, and blood glucose level in the morning. Conversely, CRY overexpression resulted in decreased glucose production in *db/db* mice, a mouse model of obesity and diabetes with excess gluconeogenesis (40). It is difficult to argue that a similar mechanism may be responsible for the phenotype of *Cry1*^−/−^ mice in the current study as their blood glucose levels were not significantly different from those of WT animals when fed a HFD. Clearly, future studies should provide further insight into the underlying mechanism of the resistance of *Cry1*^−/−^ mice to weight. It will be important to determine whether the metabolism profile of *Cry1* deficiency is the result of disruption of the circadian regulation of metabolism and/or involves transcriptional mechanisms by which Cry regulates metabolic gene expression.

In summary, these results highlight further the importance of CRY as an important regulator of energy homeostasis by showing that ablation of *Cry1*^−/−^, but not *Cry2*^−/−^ is associated with relative resistance to HFD-induced obesity. While our data suggest that *Cry1* may be a potential novel therapeutic target to combat diet-induced obesity, future studies will be needed to determine if a pharmacological manipulation of *Cry1* also prevents weight gain.

## Conflict of Interest Statement

The authors declare that the research was conducted in the absence of any commercial or financial relationships that could be construed as a potential conflict of interest.
